# Draft genome sequence of antibiotic-resistant *Shigella* *flexneri* MTR_GR_V146 strain isolated from a tomato (*Solanum lycopersicum*) sample collected from a peri-urban area of Bangladesh

**DOI:** 10.1128/mra.00099-24

**Published:** 2024-02-27

**Authors:** Md. Saiful Islam, Pritom Kumar Pramanik, Md. Liton Rana, Md. Ashek Ullah, Fahim Haque Neloy, Srinivasan Ramasamy, Pepijn Schreinemachers, Ricardo Oliva, Md. Tanvir Rahman

**Affiliations:** 1 Department of Microbiology and Hygiene, Faculty of Veterinary Science, Bangladesh Agricultural University, Mymensingh, Bangladesh; 2 Department of Animal Sciences, University of California—Davis, Davis, California, USA; 3 World Vegetable Center, Shanhua, Tainan, Taiwan; 4 World Vegetable Center – East and Southeast Asia, Bangkok, Thailand; University of Maryland School of Medicine, Baltimore, Maryland, United States

**Keywords:** vegetables, gardening systems, *Shigella flexneri*, draft genome, ARGs, VFGs, public health, Bangladesh

## Abstract

This study announces the genome sequence of the *Shigella flexneri* MTR_GR_V146 strain isolated from a tomato (*Solanum lycopersicum*) sample in Bangladesh. This strain has a 4,624,521 bp genome length (coverage: 73.07×), 2 CRISPR arrays, 1 plasmid, 52 predicted antibiotic resistance genes, and 53 virulence factor genes.

## ANNOUNCEMENT

The global emergence of foodborne pathogens has become a major concern for public health (1). The presence of antibiotic-resistant *Shigella* spp. in vegetables presents a public health risk as it can transfer to the human population through the food supply chain.

From September 2022 to March 2023, samples of fresh tomatoes (*Solanum lycopersicum*) were collected from various gardening systems in the Gazipur district of Bangladesh (24.0958°N, 90.4125°E) and subsequently transported to the laboratory at Bangladesh Agricultural University (24.7245°N, 90.4372°E). As previously described (2), the samples were aseptically cut, measured (50 g), and introduced into a sterile polyethylene stomacher bag with 200 mL of buffered peptone water. Samples were macerated for 5 minutes at 230 rpm using a Stomacher 400 circulator (Seward Ltd., London, UK). The processed samples were then incubated at 37°C for 24 h, spread on Salmonella-Shigella agar (HiMedia, Mumbai, Maharashtra, India) plates, and incubated at 37°C overnight. The resulting large, circular, convex, and transparent colonies were subjected to Gram staining and biochemical tests (oxidase, urease, carbohydrate fermentation test or mannitol, and H_2_S tests) to isolate *Shigella* spp. (3). In this study, the MTR_GR_V146 strain was chosen and incubated in nutrient broth (HiMedia, Mumbai, Maharashtra, India) at 37°C overnight. DNA was subsequently extracted from the cultured broth using a DNeasy Blood and Tissue Kit (QIAGEN, Hilden, Germany). The DNA library was prepared using the Nextera DNA Flex Library Prep Kit (Illumina, San Diego, CA, USA). Genome sequencing was carried out on the Illumina NextSeq2000 platform, which generated paired-end reads with a length of 2 × 150 bp. The genome assembly was conducted using Unicycler.v0.4.9 (4), following a preliminary step of trimming the raw paired-end reads (pre-trimming: 2,806,842; post-trimming: 2,753,822) with Trimmomatic.v0.39 (5) (leading: 20, sliding window: 4:20:20, trailing: 20, and minlen = 36) to remove Illumina adapters and phiX reads from the data set. Quality assessment was performed using FastQC.v0.11.7 (6). The SpeciesFinder.v2.0 (7) was used to identify *Shigella flexneri*. The annotation of the genome was done using PGAP.v6.6 (8). In the assembled genome, pathogenicity index by PathogenFinder.v1.1 (9); CRISPR arrays and prophages by CRISPRimmunity (10); plasmids by PlasmidFinder.v2.1 (11); antibiotic resistance genes (ARGs) by CARD.v3.2.4 with the Resistance Gene Identifier(RGI) main (12); and virulence factor genes (VFGs) by VFDB with VFanalyzer (13). Default parameters were used for all software unless otherwise specified.

In SpeciesFinder, the isolate was identified as *S. flexneri*. The assembled *S. flexneri* MTR_GR_V146 strain had 89 contigs, a GC content of 50.8%, and eight L50 contigs with a value of 182,395 bp for N50. The overall genome size was 4,624,521 bp with a coverage of 73.07×. The genome featured 4,514 genes, 4,423 CDS, 91 RNA genes (tRNAs – 76, rRNAs – 5, and non-coding RNAs – 10), 110 pseudogenes, 2 CRISPR arrays with 10 genes (*csa3*, *cas2*, *cas1*, *cas6e*, *cas5*, *cas7*, *cse2gr11*, *cas8e*, *cas3*, and *WYL*), 10 prophages, and 1 plasmid [IncFIB(AP001918)]. The genome exhibited a pathogenicity index of 0.943. Additionally, 52 predicted ARGs and 53 predicted VFGs were identified in the genome.

## Data Availability

The WGS shotgun analysis of *S. flexneri* MTR_GR_V146 was deposited to GenBank under the accession number JAVTVS000000000. The relevant data, including the raw reads, were submitted with BioProject accession number PRJNA1019910, BioSample accession number SAMN37503358, and SRA accession number SRR26156559. In this version, the specific version being referred to is identified as JAVTVS000000000.1.
